# Solid-State Synthesis and Thermoelectric Properties of CuFeSe_2_–CuFeS_2_ Solid Solutions

**DOI:** 10.3390/ma18061366

**Published:** 2025-03-19

**Authors:** Soon-Man Jang, Il-Ho Kim

**Affiliations:** Department of Materials Science and Engineering, College of Engineering, Korea National University of Transportation, Chungju 27469, Republic of Korea; soonman0118@naver.com

**Keywords:** thermoelectric, eskebornite, chalcopyrite, solid solution, composite

## Abstract

Thermoelectric technology, which converts heat and electricity into each other, has been attracting attention from the perspective of efficient energy utilization. Recently, eco-friendly and cost-effective Cu-based thermoelectric materials have been actively studied. In particular, efforts have been made to improve thermoelectric properties and enhance performance through the formation of solid solutions. This study examines the formation and thermoelectric properties of Cu-chalcogenide solid solutions between eskebornite (tetragonal CuFeSe_2_) and chalcopyrite (tetragonal CuFeS_2_), synthesized as CuFeSe_2−y_S_y_ (y = 0–2) using solid-state synthesis. These compounds share similar crystal structures, which enable the formation of solid solutions that enhance phonon scattering and may potentially improve thermoelectric performance. As the S content (y) increased, the lattice parameters *a* and *c* decreased, attributed to the smaller ionic radius of S^2−^ compared to Se^2−^, as X-ray diffraction analysis identified single-phase regions for 0 ≤ y ≤ 0.4 and 1.6 ≤ y ≤ 2, respectively. However, for 0.8 ≤ y ≤ 1.2, a composite phase of eskebornite and chalcopyrite formed, indicating incomplete solid solution behavior in the intermediate range. Thermoelectric measurements showed a sharp increase in electrical conductivity with increasing S content, alongside a transition in the Seebeck coefficient from positive (p-type) to negative (n-type), attributed to the intrinsic semiconducting nature of the end-member compounds. Eskebornite behaves as a p-type semiconductor, whereas chalcopyrite is n-type, and their combination affects the carrier type and concentration. Despite these changes, the power factor did not show significant improvement due to the inverse relationship between electrical conductivity and the Seebeck coefficient. The thermal conductivity decreased significantly with solid solution formation, with CuFeSe_0.4_S_1.6_ exhibiting the lowest value of 0.97 Wm^−1^K^−1^ at 623 K, a result of enhanced phonon scattering at lattice imperfections and the mass fluctuation effect. This value is lower than the thermal conductivity values of single-phase eskebornite or chalcopyrite. However, the reduction in thermal conductivity was insufficient to compensate for the modest power factor, resulting in no substantial enhancement in the thermoelectric figure of merit.

## 1. Introduction

Enhancing thermoelectric performance requires a combined focus on increasing the power factor and reducing thermal conductivity. The power factor, defined as the product of the square of the Seebeck coefficient and electrical conductivity, can be optimized by balancing these two properties, which are often inversely related. Reducing thermal conductivity involves minimizing lattice thermal conductivity by enhancing phonon scattering mechanisms, which can be effectively achieved through strategies such as forming substitutional solid solutions or introducing structural heterogeneities. The formation of substitutional solid solutions is widely recognized as an effective approach to improving thermoelectric performance. This method enhances phonon scattering by introducing mass and strain fluctuations in the crystal lattice, thereby reducing lattice thermal conductivity. Additionally, such solutions enable fine-tuning of carrier concentration through band gap modifications, providing an opportunity to optimize electronic transport properties [1]. Lee [2] presented an exemplary case with Bi_2_Te_3_–Bi_2_Se_3_ solid solutions (Bi_2_Te_3−y_Se_y_), where substituting Se for Te induced lattice distortion, enhancing phonon scattering and significantly lowering lattice thermal conductivity. This structural modification resulted in an overall improvement in thermoelectric performance. Similarly, Zhang et al. [3] demonstrated that forming Bi_2_Te_3_–Bi_2_S_3_ composites exhibited remarkable thermoelectric properties over a broad temperature range. The enhancement was primarily attributed to reduced thermal conductivity achieved through controlled carrier concentration and enhanced phonon scattering at the grain boundaries of the composite structure.

Cu-chalcogenide materials, including eskebornite (CuFeSe_2_) and chalcopyrite (CuFeS_2_), have attracted attention as eco-friendly thermoelectric materials due to their cost-effectiveness, abundance, and low toxicity [4,5,6]. Eskebornite, a p-type semiconductor with the tetragonal $P\bar{4}2c$ space group [7], and chalcopyrite, an n-type semiconductor with the tetragonal $I\bar{4}2d$ space group [8], share the same I–III–VI_2_ chemical composition. Their structural compatibility and the similar ionic radii of Se^2−^ (198 pm) and S^2−^ (184 pm) [9] enable the formation of solid solutions through anion substitution. This leads to lattice distortions, changes in band gap energy, and transitions between p- and n-type conduction, providing opportunities for tailored thermoelectric properties. Several studies have highlighted the impact of such substitutions on thermoelectric performance. Skoug et al. [10] investigated Cu_3_SbSe_4−x_S_x_ solid solutions, where permingeatite Cu_3_SbSe_4_ and famatinite Cu_3_SbS_4_ form a tetragonal solid solution. They found that the lattice thermal conductivity decreased with increasing S content due to enhanced phonon scattering from the disordered arrangement of Se and S atoms. At x = 0.5, the experimentally observed lattice thermal conductivity was 1.75 Wm^−1^K^−1^, lower than the theoretical minimum value of 1.91 Wm^−1^K^−1^ predicted by the Debye model. This reduction was attributed to additional phonon scattering caused by the anion disorder. Similarly, Skoug et al. [11] reported that in Ge-doped Cu_3_Sb_1−y_Ge_y_Se_4−x_S_x_ solid solutions, the lattice thermal conductivity approached the theoretical minimum due to disordered anion arrangements, further emphasizing the effectiveness of substitutional solid solutions in reducing lattice thermal conductivity. Lee and Kim [12] explored the thermoelectric properties of Cu_3_SbSe_4−y_S_y_ (y = 0−4) and observed that increasing S content reduced electrical conductivity due to lower carrier concentration and mobility. However, the Seebeck coefficient increased slightly, enhancing the power factor. Simultaneously, the lattice thermal conductivity decreased significantly, resulting in a noticeable reduction in total thermal conductivity. Despite these improvements, the thermoelectric figure of merit did not improve substantially due to limited gains in the power factor. This aligns with observations by Carr and Morelli [5], who studied CuFeS_2(1−x)_Se_2x_ solid solutions. They reported a significant reduction in electrical resistivity with increasing Se content, leading to an improved power factor compared to CuFeS_2_. However, the Seebeck coefficient decreased modestly, and changes in thermal conductivity were minimal, resulting in only a marginal increase in thermoelectric figure of merit.

In our previous studies, pure eskebornite [7] and chalcopyrite [8] were successfully synthesized via mechanical alloying. The powders were subsequently hot-pressed for phase analysis, microstructural observations, charge transport measurements, and thermoelectric property evaluations. This approach enabled a comprehensive understanding of the materials’ phase stability and thermoelectric behavior. Building on this foundation, the present study investigates the impact of solid solution formation (CuFeSe_2−y_S_y_) between eskebornite and chalcopyrite on their thermoelectric properties. Specifically, the solubility limits of Se and S in these two Cu-chalcogenide materials were investigated, focusing on how these elements influence phase stability and whether secondary phases form during solid solution synthesis. Thermal analysis was conducted to evaluate phase transformations and solubility limits, and the temperature-dependent thermoelectric properties were systematically assessed.

## 2. Experimental Procedure

To synthesize CuFeSe_2−y_S_y_ with varying stoichiometric compositions (y = 0, 0.4, 0.8, 1.2, 1.6, and 2), high-purity elemental powders of Cu (99.9%, <45 μm), Fe (99.9%, <53 μm), Se (99.999%, <10 μm), and S (99.99%, <75 μm) were weighed based on their stoichiometric ratios. The powders were initially mixed using a ball mill (PL-BM5L, PLTECH, Seoul, Republic of Korea) for 1 h to ensure uniform distribution. The mixed powders were subsequently subjected to mechanical alloying (MA; Pulverisette5, Fritsch, Idar-Oberstein, Germany) in an argon atmosphere to prevent oxidation. The mechanically alloyed powders were consolidated using hot pressing (HP; JM-HP20, Jungmin, Seoul, Republic of Korea) at a pressure of 70 MPa under vacuum. The HP temperature was varied between 623 K and 773 K for 2 h, following previously optimized conditions for CuFeSe_2_ [7] and CuFeS_2_ [8]. These conditions produced the most well-consolidated samples when MA was performed at 350 rpm for 12 h, with HP at 623 K for CuFeSe_2_ and 773 K for CuFeS_2_.

For phase analysis and thermoelectric property evaluation, sintered samples were cut into appropriately sized pieces using a precision diamond saw (ISOMET, Buehler, Lake Bluff, IL, USA) to ensure smooth and precise sectioning. The mechanically alloyed powders and hot-pressed specimens were analyzed using X-ray diffraction (XRD; D8-Advance, Bruker, Billerica, MA, USA) with Cu Kα radiation. XRD was used to evaluate the formation of solid solutions and to perform detailed phase analysis. Lattice constants and crystallite sizes were determined through Rietveld refinement of the XRD data using the TOPAS software (v4.1, Bruker, Billerica, MA, USA). Thermal analysis was conducted to investigate the thermal behavior and phase transitions of the samples. Thermogravimetric (TG) and differential scanning calorimetry (DSC) measurements were conducted using a TG/DSC1 instrument (Mettler Toledo, Columbus, OH, USA) in an argon atmosphere at a heating rate of 5 K/min. Polished specimen surfaces were analyzed using scanning electron microscopy (SEM; Prisma E, Thermo Fisher Scientific, Waltham, MA, USA) in backscattered electron (BSE) mode to investigate overall morphology and phase distribution. Fractured specimen surfaces were observed in secondary electron (SE) mode to analyze microstructural features. Compositional analysis was performed using energy-dispersive spectroscopy (EDS; Quantax200, Bruker, Billerica, MA, USA).

The Seebeck coefficient (α) and electrical conductivity (σ) of the samples were measured using a DC four-probe method with a ZEM-3 system (Advance Riko, Yokohama, Japan) under a helium atmosphere. The power factor (PF = α^2^σ) was subsequently calculated from on the measured values. Thermal diffusivity (D) was measured using the laser flash method with a TC-9000H system (Advance Riko, Yokohama, Japan) to evaluate the thermal properties. Thermal conductivity (κ) was determined using the relation κ = D ∙ d ∙ c_p_, where d is the material density and c_p_ is its specific heat. Finally, the dimensionless figure of merit (ZT) was calculated using the formula ZT = PF ∙ κ^−1^ ∙ T. The ZT values were evaluated over a temperature (T) range of 323–623 K. 

## 3. Results and Discussion

**Figure 1:**
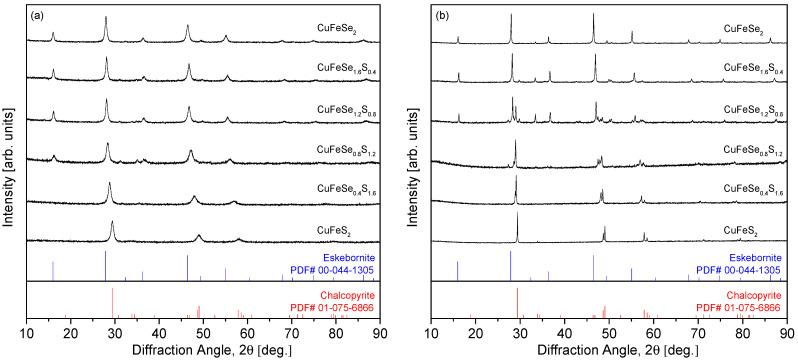
XRD patterns for the (**a**) MA powders and (**b**) HP specimens of CuFeSe_2−y_S_y_.

Figure 1 shows the XRD patterns of the MA powders and HP compacts for the CuFeSe_2−y_S_y_ samples. As depicted in Figure 1a, for the MA powders, the diffraction peaks shift toward higher angles as the S content (y) increases. This shift indicates the formation of a substitutional solid solution between the two end-member compounds, eskebornite (CuFeSe_2_) and chalcopyrite (CuFeS_2_). The absence of secondary phases in the MA powders suggests that S substitution in the solid solution is uniformly distributed across the compositions. This is consistent with the findings of Carr and Morelli [5], who observed similar behavior in their study of CuFeS_2(1−x)_Se_2x_ solid solutions, where the diffraction peaks shifted to lower angles as Se content increased, with the formation of mixed phases at higher S content (x = 0.3). However, in the CuFeS_2(1−x)_Se_2x_ series, secondary phases such as bornite (Cu_5_FeS_4_) and covellite (CuS) appeared during the milling process, but were eliminated after HP, yielding a single-phase chalcopyrite solid solution. In contrast, Figure 1b shows that after HP, phase separation occurs between eskebornite and chalcopyrite in the y = 0.8–1.2 compositions, resulting in a composite phase. This behavior contrasts with the findings of Skoug et al. [10], who studied Cu_3_SbSe_4−x_S_x_ solid solutions and reported no phase separation in the mechanically alloyed powders. They observed only slight changes in lattice constants and thermal conductivity due to sulfur substitution. Furthermore, our previous study [12] on permingeatite–famatinite solid solutions (Cu_3_SbSe_4−y_S_y_) supports the reliability of the MA–HP approach, as no secondary phases were observed in the solid solutions despite varying sulfur content. 

**Figure 2:**
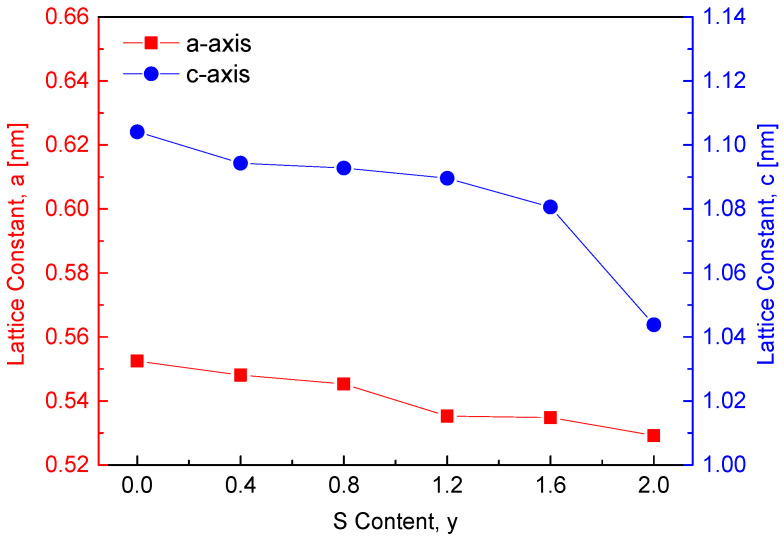
Variations in the lattice constants of the CuFeSe_2−y_S_y_.

Figure 2 depicts the variation in lattice constants with sulfur content in the CuFeSe_2−y_S_y_ solid solution series. The lattice constants of the two end-member compounds, CuFeSe_2_ (y = 0) and CuFeS_2_ (y = 2), are reported as follows: CuFeSe_2_ (a = 0.5525 nm, c = 1.1041 nm, c/a = 1.9984) [7] and CuFeS_2_ (a = 0.5292 nm, c = 1.0438 nm, c/a = 1.9724) [8]. As expected, substituting the larger Se^2−^ ions (ionic radius: 198 pm) with smaller S^2−^ ions (ionic radius: 184 pm) [9] reduces both the a and c lattice constants. In comparison, Carr and Morelli [5] observed an opposite trend in CuFeS_2(1−x)_Se_2x_ solid solutions, where increasing Se content (x) resulted in a linear increase in lattice parameters. This is likely attributed to the larger ionic radius of Se^2−^ compared to S^2−^, which expands the unit cell. However, when the Se content reached 0.3 (i.e., CuFeS_1.4_Se_0.6_), a decrease in lattice constants was observed due to the formation of a secondary phase, FeSe. This suggests that introducing excess Se in CuFeS_2(1−x)_Se_2x_ or excess S in CuFeSe_2−y_S_y_ at higher concentrations may destabilize the solid solution, resulting in phase separation and reduced lattice constants.

**Figure 3:**
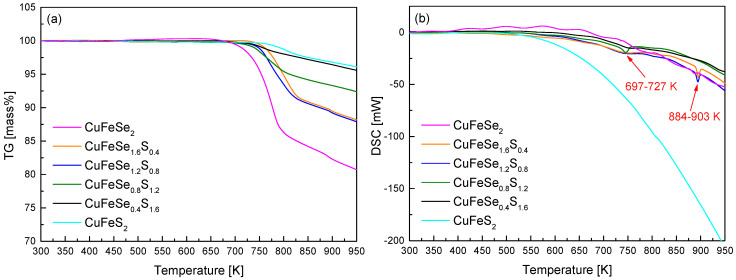
Thermal analyses of (**a**) TG and (**b**) DSC for CuFeSe_2−y_S_y_ prepared via the MA–HP process.

Figure 3 presents the thermal analysis results for CuFeSe_2−y_S_y_. The TG curves (Figure 3a) show a clear trend where the temperature at which mass loss initiates increases with higher sulfur content. For CuFeSe_2_ (y = 0), mass loss begins at 675 K, while for CuFeS_2_ (y = 2), mass loss starts at a significantly higher temperature of 790 K. This shift in the onset of mass loss suggests that the sulfur substitution enhances the thermal stability of chalcopyrite relative to eskebornite. In addition to the increase in the initiation temperature of mass loss, the TG curves also indicate that the rate of mass loss decreases with increasing S content. The gentler slopes observed in the TG curves of chalcopyrite compared to eskebornite further support the conclusion that chalcopyrite is more thermally stable at elevated temperatures. This suggests that sulfur substitution may strengthen the crystal lattice of chalcopyrite, making it less prone to thermal degradation or phase transition under high-temperature conditions. A closer examination of the mass loss behavior reveals that it is associated with the melting and volatilization of constituent elements, particularly chalcogen elements of Se and S. The higher onset temperature for mass loss in chalcopyrite is indicative of the higher stability of S within the structure compared to Se, which has a lower volatility. The volatilization of chalcogen elements is a critical factor influencing the stability and phase integrity of both eskebornite and chalcopyrite.

In the DSC plots (Figure 3b), two endothermic peaks were observed at temperatures of 697–727 K and 884–903 K, with the higher-temperature endothermic peaks shifting to higher temperatures as the S content increased. The melting point of CuFeSe_2_ is reported as 850 K [13], whereas CuFeS_2_ exhibits a higher melting point, ranging from 1120 K to 1150 K [14]. A detailed analysis of the DSC data reveals the following temperature ranges for the endothermic peaks: y = 0 at 740–749 K; y = 0.4 at 697–741 K and 886–890 K; y = 0.8 at 697–738 K and 884–889 K; y = 1.2 at 728–742 K; y = 1.6 at 713–746 K; y = 2 at 805–813 K and 891–895 K. The endothermic peak in the 697–727 K range is attributed to phase transitions occurring at lower S contents, while the peak in the 884–903 K range is associated with additional phase transitions at higher S contents and temperatures. This suggests that increasing the S content promotes the formation of solid solutions, wherein Se is substituted by S, resulting in a higher melting point or phase transition temperature. Choi and Kim [7] reported that pure eskebornite undergoes rapid mass loss above 823 K due to Se volatility, with endothermic peaks at 805–813 K and 891–895 K, confirming its stability up to approximately 750 K. Kim and Kim [8] demonstrated that in pure chalcopyrite, the endothermic peak at 740–749 K corresponds to its synthesis from residual elements, whereas the larger peak at 1169–1170 K is associated with its melting point. Carr and Morelli [5] found that CuFeS_2_ decomposes at 820 K due to sulfur loss, resulting in the formation of FeS_2_, CuS, and FeS, and that the decomposition temperature decreased to 750 K when the Se replaced S. This reduction is likely due to the larger ionic radius of Se, which leads to longer bond lengths. Lee and Kim [12] also observed that in Cu_3_SbSe_4−y_S_y_, increasing the S content raises the melting point, with mass loss occurring above the melting point due to the volatilization of chalcogen elements.

**Figure 4:**
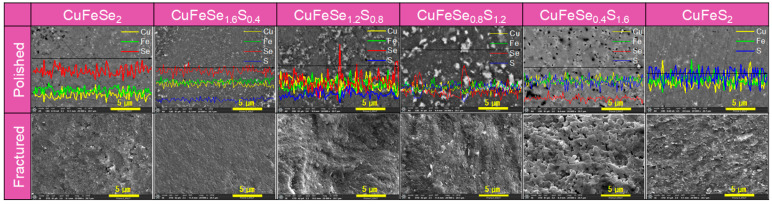
SEM images of polished and fractured surfaces for CuFeSe_2−y_S_y_.

The microstructural and compositional analysis of the CuFeSe_2−y_S_y_ sintered samples, presented in Figure 4, offers a detailed understanding of their structural evolution with increasing S content. SEM observations and XRD refinements revealed a significant decrease in the average crystallite size, from 63 nm at lower S content to 46 nm at higher S content. The crystallite size was determined from XRD data using Rietveld refinement. Theoretical density values for CuFeSe_2_ and CuFeS_2_ are reported as 5.35 gcm^−3^ [13] and 3.19 gcm^−3^ [14], respectively. By applying the rule of mixture, the calculated relative densities of CuFeSe_2−y_S_y_ exceeded 97.4%, confirming that the samples were highly dense with minimal porosity. EDS elemental analysis confirmed that the elemental compositions closely aligned with the nominal values within an acceptable margin of error. As the S content increased, the EDS spectra revealed a corresponding rise in S peak intensity and a decline in Se peak intensity, consistent with the substitution of Se by S within the lattice. However, in the samples with y = 0.8 and 1.2, distinct bright regions were observed, showing a sharp increase in Se peak intensity accompanied by a decrease in S intensity. These findings indicate phase separation, with Se-enriched regions coexisting alongside the primary CuFeSe_2−y_S_y_ phase. This interpretation is consistent with the XRD results shown in Figure 1b, which reveal secondary phase formation for these specific compositions.

**Figure 5:**
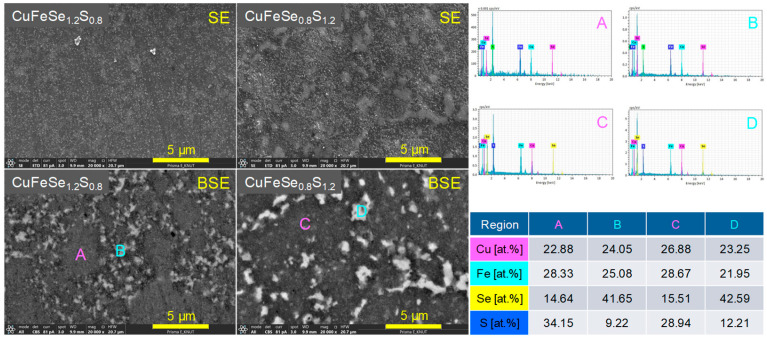
EDS analyses of CuFeSe_1.2_S_0.8_ and CuFeSe_0.8_S_1.2_, indicating the phase separations.

Figure 5 shows the SEM images and EDS elemental spot analyses of the CuFeSe_1.2_S_0.8_ and CuFeSe_0.8_S_1.2_ samples, emphasizing their microstructural and compositional features. In secondary electron (SE) mode, both samples displayed smooth surfaces and dense microstructures, indicating effective sintering with minimal porosity. However, in backscattered electron (BSE) mode, distinct regions with varying contrast were observed, indicating heterogeneity in chemical composition. EDS analyses confirmed that the dark regions (A and C) were enriched in sulfur, forming S-rich solid solutions, while the bright regions (B and D) were enriched in selenium, representing Se-rich solid solutions. This observation indicates that for compositions in the range of 0.8 ≤ y ≤ 1.2, phase separation occurred, leading to the coexistence of two distinct solid solutions. Such phase separation likely arises from the limited solubility of Se and S in the CuFeSe_2−y_S_y_ system within this compositional range.

**Figure 6:**
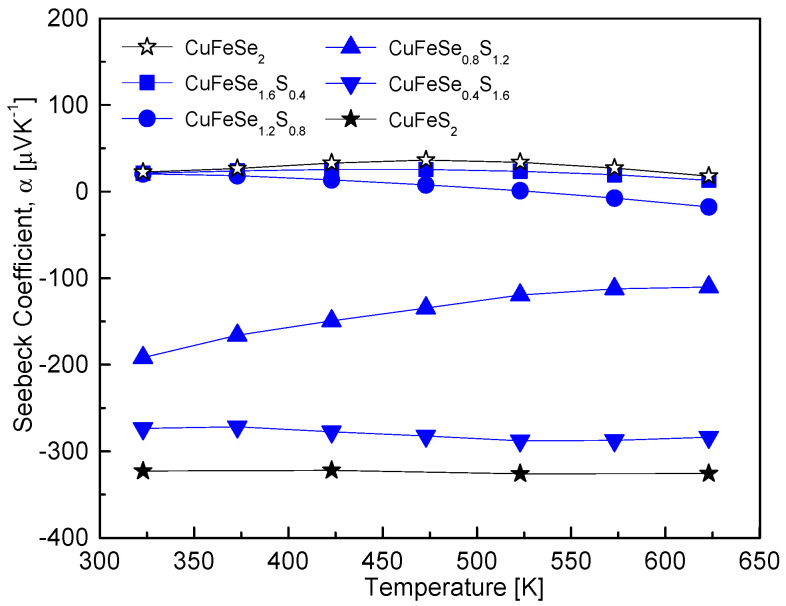
Temperature-dependent variation of the Seebeck coefficient for CuFeSe_2−y_S_y_ samples, indicating the influence of sulfur substitution on Seebeck coefficient behavior.

Figure 6 illustrates the temperature-dependent Seebeck coefficient of CuFeSe_2−y_S_y_, showing a conduction type transition from p-type (positive values) to n-type (negative values) with increasing S content. This behavior corresponds to the intrinsic nature of CuFeSe_2_ as a p-type semiconductor and CuFeS_2_ as an n-type semiconductor. The temperature at which the Seebeck coefficient transitions also depended on the S content: for y = 0–0.4, the transition occurred around 473 K; for y = 0.8, it was observed at 373 K; and for y = 1.2, it shifted below 323 K. Notably, for y = 1.6–2, the Seebeck coefficient exhibited minimal temperature dependence, indicating stabilized n-type behavior over the temperature range. The variations in the Seebeck coefficient are closely related to changes in the electronic structure of the CuFeSe_2−y_S_y_ solid solutions. The substitution of Se with S increases the band gap due to sulfur’s higher electronegativity and smaller ionic radius, which alters the electronic band structure. Specifically, CuFeSe_2_ has a reported band gap of 0.16 eV [15], whereas CuFeS_2_ exhibits a larger band gap of 0.53 eV [5]. As the S content increases, the widening band gap leads to a reduction in carrier concentration, which initially contributes to an increase in the Seebeck coefficient. This trend aligns with observations in other solid solutions, such as Cu_3_SbSe_4−y_S_y_, where S substitution similarly widened the band gap, reduced the carrier concentration, and enhanced the Seebeck coefficient [12]. However, the behavior of CuFeSe_2−y_S_y_ becomes more complex due to the p–n transition with increasing S content. As the material transitions from p-type to n-type, the majority carriers shift from holes to electrons, leading to significant changes in the Seebeck coefficient. For samples with higher S content (y = 1.5–2), the behavior matches findings by Carr and Morelli [5], who reported that CuFeS_2(1−x)_Se_2x_ solid solutions (x = 0–0.25, corresponding to y = 1.5–2) consistently exhibited n-type Seebeck coefficients. They observed intrinsic transitions around 400 K, with Seebeck coefficients reaching a maximum between −400 μVK^−1^ and −500 μVK^−1^. At elevated temperatures (e.g., 670 K), the Seebeck coefficient of CuFeS_1.5_Se_0.5_ decreased to −303 μVK^−1^, indicating that increased carrier concentrations at high temperatures suppress the Seebeck coefficient. 

**Figure 7:**
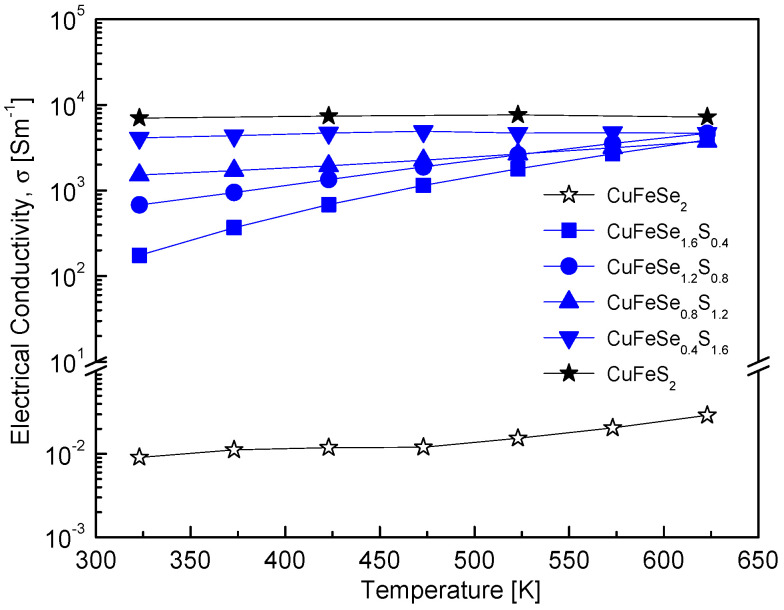
Temperature-dependent variation of the electrical conductivity for CuFeSe_2−y_S_y_ samples, indicating the influence of sulfur substitution on electrical conductivity behavior.

Figure 7 shows the electrical conductivity of CuFeSe_2−y_S_y_ as a function of temperature and S content, highlighting a significant increase in electrical conductivity with higher S content. For example, at 323 K, the electrical conductivity increased from 9.1 × 10^−3^ Sm^−1^ for y = 0 to 7.0 × 10^3^ Sm^−1^ for y = 2. Additionally, the electrical conductivity exhibited a positive temperature dependence, increasing with rising temperature. However, this dependence weakened as the S content increased. The observed trend of increasing electrical conductivity with S substitution is intriguing because Se^2−^ and S^2−^ are isovalent ions, and CuFeSe_2_ and CuFeS_2_ have different band gaps (0.16 eV for CuFeSe_2_ [15] and 0.53 eV for CuFeS_2_ [5]). Intuitively, substituting S for Se would be expected to increase the band gap and reduce electrical conductivity. This discrepancy is likely caused by the interplay between the p–n transition and carrier concentration. CuFeSe_2_, being a p-type semiconductor, predominantly conducts via holes, while CuFeS_2_, as an n-type semiconductor, conducts via electrons. As the S content increases, the majority carrier type shifts from holes to electrons, fundamentally altering the conduction mechanism and band gap energy, which makes it difficult to explain the electrical conductivity trends based solely on band gap differences. This phenomenon is comparable to observations by Carr and Morelli [5] in CuFeS_2(1−x)_Se_2x_, where increasing Se content reduced electrical resistivity (and increased conductivity) by up to an order of magnitude at 250 K and approximately twofold at 670 K. They attributed this to band gap narrowing due to Se substitution. Similarly, Lee and Kim [12] reported in Cu_3_SbSe_4−y_S_y_ that increasing S content enhanced the temperature dependence of electrical conductivity, but reduced conductivity at certain temperatures. This reduction was attributed to a decline in mobility and carrier concentration. However, both studies focused on p-type systems without p–n transitions, making their interpretation simpler compared to the current study. In the present study, the intrinsic electrical properties of eskebornite (CuFeSe_2_) and chalcopyrite (CuFeS_2_) must also be considered. Intrinsic chalcopyrite exhibits significantly higher electrical conductivity than intrinsic eskebornite. Therefore, as the proportion of chalcopyrite-like material increases with S substitution, the electrical conductivity of the resulting solid solution or composite improves. This contribution of the chalcopyrite phase offers an additional explanation for the increasing conductivity observed with higher S content. Moreover, the p–n transition and corresponding changes in carrier type further amplify this effect, distinguishing the behavior of CuFeSe_2−y_S_y_ from that of purely p-type systems.

**Figure 8:**
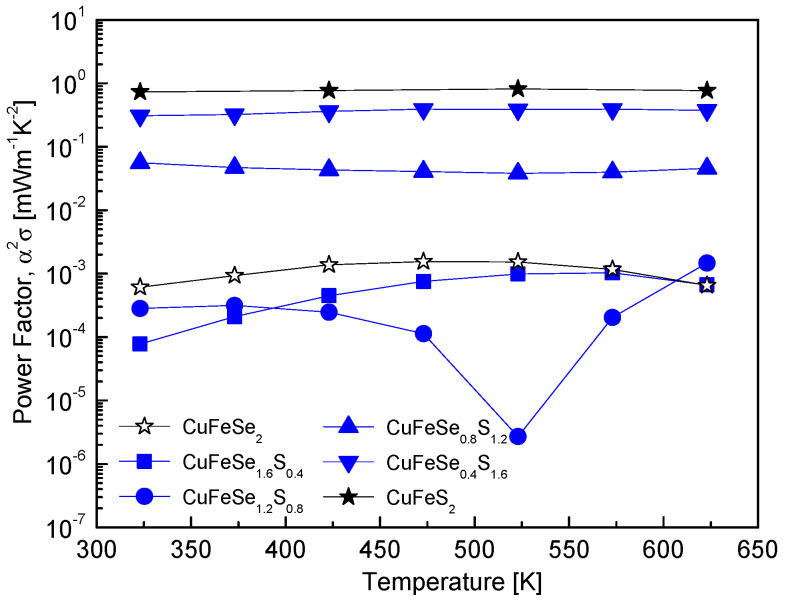
Temperature-dependent variation of the power factor for CuFeSe_2−y_S_y_ samples, indicating the influence of sulfur substitution on power factor behavior.

Figure 8 illustrates the power factor of CuFeSe_2−y_S_y_, showing a significant enhancement with increasing S content. This trend is attributed to the intrinsic properties of its constituent phases. Pure chalcopyrite exhibits a significantly higher power factor (0.81 mWm^−1^K^−2^ at 523 K) than pure eskebornite (1.5 μWm^−1^K^−2^ at 473 K). Consequently, as the S content increases and the material transitions toward a chalcopyrite-dominant composition, the power factor of the solid solutions increases significantly. This behavior contrasts with the findings of Carr and Morelli [5] in the CuFeS_2(1−x)_Se_2x_ system. They reported that the power factor increased with Se content, peaking at 670 K with values of 0.30 mWm^−1^K^−2^ for CuFeS_2_ and 0.57 mWm^−1^K^−2^ for CuFeS_1.6_Se_0.4_. However, further increases in Se content caused a decline in the power factor, likely due to changes in the material’s electronic structure and transport properties, such as reduced carrier mobility or suboptimal carrier concentration. In CuFeSe_2−y_Sy, the sharp increase in power factor with S substitution is primarily attributed to the substantial improvement in electrical conductivity observed in Figure 7, combined with a moderate Seebeck coefficient. This combination enhances the power factor, especially in compositions dominated by the chalcopyrite phase. Additionally, the intrinsic differences in carrier concentration and mobility between the eskebornite and chalcopyrite phases further amplify the power factor in S-rich compositions.

**Figure 9:**
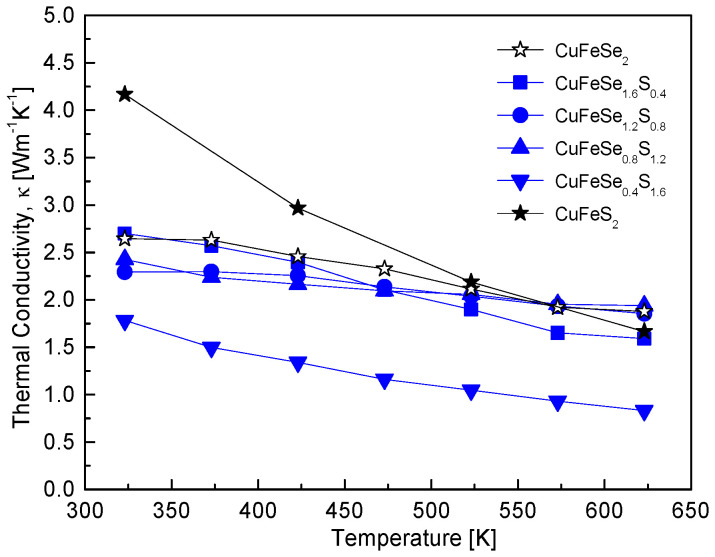
Temperature-dependent variation of the thermal conductivity for CuFeSe_2−y_S_y_ samples, indicating the influence of sulfur substitution on thermal conductivity behavior.

Figure 9 illustrates the thermal conductivity of CuFeSe_2−y_S_y_ solid solutions, emphasizing the impact of compositional tuning on phonon scattering and thermal transport. The specific heat values used for thermal conductivity calculations were estimated using the rule of mixtures, combining reported values for CuFeSe_2_ (0.344 Jg^−1^K^−1^) [16] and CuFeS_2_ (0.52 Jg^−1^K^−1^) [17]. The trend indicates a decrease in thermal conductivity with increasing temperature, consistent with typical phonon-dominated thermal transport in semiconductors. Notably, the thermal conductivity of composite specimens (0.4 ≤ y ≤ 1.6) is lower than that of the pure compounds, CuFeSe_2_ and CuFeS_2_. Among these, CuFeSe_0.4_S_1.6_ exhibits the lowest thermal conductivity, decreasing from 1.78 Wm^−1^K^−1^ at 323 K to 0.83 Wm^−1^K^−1^ at 623 K. This reduction is attributed to enhanced phonon scattering induced by alloying. The substitution of Se with S introduces mass and bond strength disparities, disrupting lattice periodicity and generating additional phonon scattering centers, thereby reducing thermal conductivity. This behavior aligns with the findings of Carr and Morelli [5], who reported a T^−1^ dependence of thermal conductivity in CuFeS_2(1−x)_Se_2x_ at temperatures above 350 K, where Umklapp scattering becomes the dominant mechanism [18,19]. However, at 670 K, the thermal conductivity values they observed (1.7–2.5 Wm^−1^K^−1^) were higher than those of other chalcopyrite materials, which typically exhibit thermal conductivity below 1 Wm^−1^K^−1^. 

**Figure 10:**
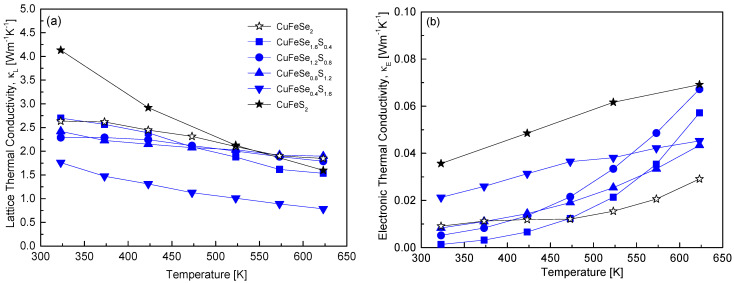
Contributions to thermal conductivity of CuFeSe_2−y_S_y_: (**a**) lattice thermal conductivity and (**b**) electronic thermal conductivity.

Figure 10 distinguishes between the lattice and electronic components of thermal conductivity in CuFeSe_2−y_S_y_ using the Wiedemann–Franz law [20]. Compared to the total thermal conductivity shown in Figure 9, phonon-mediated heat transfer, represented by lattice thermal conductivity, clearly dominates. Thus, substituting S for Se in CuFeSe_2−y_S_y_ enhances phonon scattering (via the Umklapp process), reducing lattice thermal conductivity. This observation highlights why thermoelectric materials often aim to reduce thermal conductivity by inducing lattice distortion through solid solution formation or doping. For the sample with y = 1.6, the lattice thermal conductivity reached a minimum of 0.79 Wm^−1^K^−1^ at 623 K. This value is 43–49% lower than that of pure eskebornite (1.85 Wm^−1^K^−1^ at 623 K) and pure chalcopyrite (1.60 Wm^−1^K^−1^ at 623 K). Skoug et al. [10] reported a significant decrease in the lattice thermal conductivity of Cu_3_SbSe_4−y_S_y_ (y = 0–4) with the substitution of 10% S for Se, attributed to enhanced phonon scattering from point defects, which reduced its temperature dependence. When 50% of Se was substituted, the disorder peaked, reducing the lattice thermal conductivity by 40% at 300 K compared to Cu_3_SbSe_4_. Similarly, Lee and Kim [12] reported that in Cu_3_SbSe_4−y_S_y_ (y = 0–4), Cu_3_SbSe_1.6_S_2.4_ achieved a minimum lattice thermal conductivity of 0.56 Wm^−1^K^−1^ at 623 K. This value was 84% and 78% lower than that of Cu_3_SbS_4_ (0.67 Wm^−1^K^−1^ at 623 K) and Cu_3_SbSe_4_ (0.72 Wm^−1^K^−1^ at 623 K), respectively. As illustrated in Figure 10b, the electronic thermal conductivity increased with temperature. Electronic thermal conductivity is linked to heat transfer by charge carriers, which directly depends on electrical conductivity and carrier concentration. In non-degenerate semiconductors, increasing temperature generally enhances electrical conductivity, which in turn raises electronic thermal conductivity. However, the variation in electronic thermal conductivity with S content at a given temperature was inconsistent. This inconsistency is attributed to changes in the band gap and a concurrent p–n transition as the S content increases in CuFeSe_2−y_S_y_. This transition alters the carrier type, resulting in variations in temperature-dependent bipolar conduction. 

**Figure 11:**
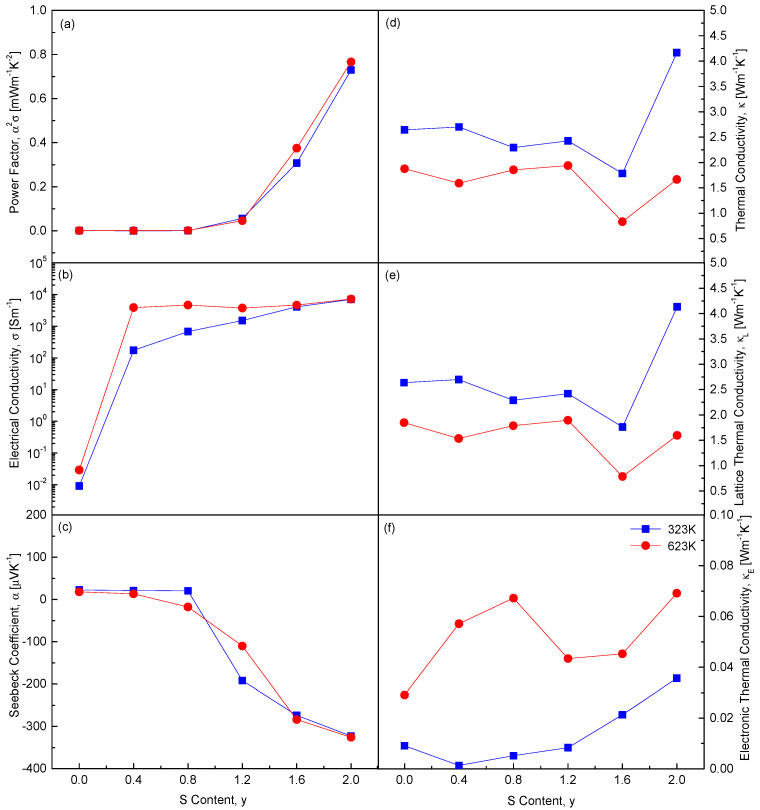
Variations of the thermoelectric parameters at 323 K and 623 K with S content for CuFeSe_2−y_S_y_: (**a**) power factor, (**b**) electrical conductivity, (**c**) Seebeck coefficient, (**d**) thermal conductivity, (**e**) lattice thermal conductivity, and (**f**) electronic thermal conductivity.

Figure 11 replots the composition-dependent thermoelectric parameters at 323 K and 623 K. As the S content (y) increases, several thermoelectric properties exhibit significant changes. Notably, both the absolute value of the Seebeck coefficient and the electrical conductivity increase. This indicates a shift in carrier concentration and type, as the Seebeck coefficient is highly sensitive to such changes. Increasing sulfur content likely alters the electronic structure, affecting both carrier concentration and charge carrier mobility. Moreover, increasing S content leads to higher electronic thermal conductivity and power factor. Electronic thermal conductivity directly correlates with electrical conductivity, as both rely on charge carriers. The power factor, defined as the product of the square of the Seebeck coefficient and electrical conductivity, also improves. Interestingly, CuFeSe_0.4_S_1.6_ exhibits the lowest lattice thermal conductivity. This reduction is attributed to enhanced phonon scattering caused by substituting Se with S. Replacing Se with S alters the atomic size and mass of the components, intensifying phonon scattering. Solid solution formation in CuFeSe_2−y_S_y_ is confirmed to be an effective strategy to minimize thermal conductivity by maximizing phonon scattering through lattice disorder, atomic substitution, and increased chemical irregularity.

**Figure 12:**
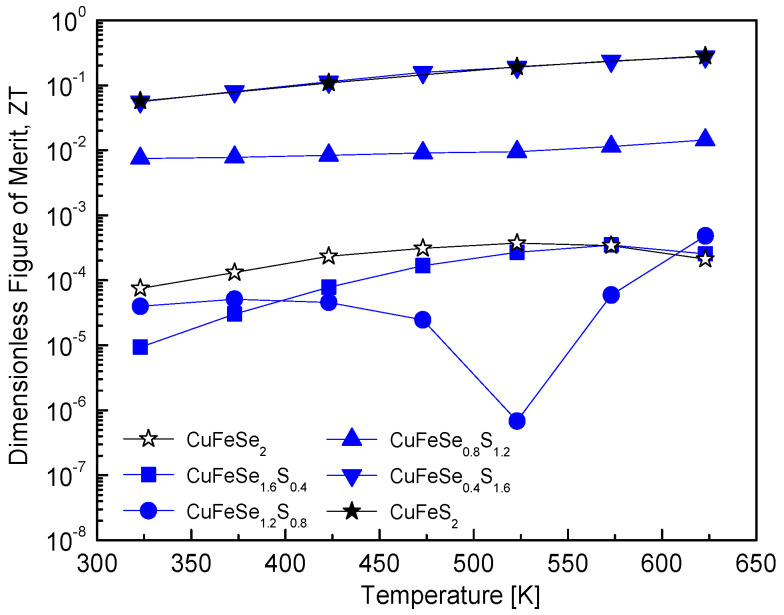
Temperature-dependent variation of the dimensionless figure of merit for CuFeSe_2−y_S_y_ samples, illustrating the influence of sulfur substitution on thermoelectric performance.

Figure 12 illustrates the ZT values of CuFeSe_2−y_S_y_, which quantify the energy conversion efficiency of thermoelectric materials. The maximum ZT value for pure eskebornite is as low as 3.73 × 10^−4^ at 523 K. This low value aligns with the inherently poor thermoelectric performance of pristine eskebornite, a material characterized by low electrical conductivity and/or high thermal conductivity. Increasing the S content to y = 1.2 results in minimal improvement in the ZT value, suggesting that S substitution does not significantly enhance thermoelectric efficiency in this composition range. However, further increasing the S content to y = 1.6 significantly improves the ZT value, reaching 2.76 × 10^−1^ at 623 K. This value is comparable to the maximum ZT of pure chalcopyrite (2.82 × 10^−1^ at 623 K). A comparison with previous studies reveals noteworthy observations. For example, Carr and Morelli [5] reported that the ZT of CuFeSe_0.1_S_1.9_, synthesized via high-energy SPEX milling and HP, increased to 0.16 at 670 K, compared to 0.09 for pure chalcopyrite. This improvement was attributed to Se substitution with S in chalcopyrite, which effectively maintained low thermal conductivity and enhanced the power factor. Despite these improvements, the ZT values reported in their study remained lower than that of CuFeSe_0.4_S_1.6_ in this study, demonstrating the superior effectiveness of the solid solution studied here in enhancing ZT. In a previous study by Lee and Kim [12], the ZT value of Cu_3_SbS_4−y_Se_y_, synthesized via the MA–HP process, increased with higher Se content but did not surpass that of pure permingeatite. However, Skoug et al. [11] demonstrated that Cu_3_Sb_1−y_Ge_y_Se_4−x_S_x_, synthesized via melting–quenching–annealing followed by the HP process, showed a significant reduction in lattice thermal conductivity due to double substitutions of Ge and S, leading to an increased ZT value. 

## 4. Conclusions

This study investigates the effects of sulfur substitution in CuFeSe_2−y_S_y_ (y = 0–2) solid solutions, composed of eskebornite (CuFeSe_2_) and chalcopyrite (CuFeS_2_), on phase behavior, microstructure, thermal stability, and thermoelectric properties. Materials were synthesized via mechanical alloying and hot pressing, with no secondary phases detected in as-milled powders. However, during hot pressing, phase separation occurred for y = 0.8–1.2, forming Se-rich eskebornite and S-rich chalcopyrite, while single-phase solid solutions formed at y ≤ 0.4 and y ≥ 1.6. This suggests solid solution stability at low and high sulfur concentrations, but phase separation at intermediate levels. Lattice constants decreased with increasing sulfur content, confirming effective substitution of sulfur for selenium. The sulfur-induced p–n transition significantly influenced the Seebeck coefficient by altering the band gap and electron concentration, thereby increasing its absolute value. Enhanced electron transport and increased Seebeck coefficient resulted in a higher power factor. CuFeSe_0.4_S_1.6_ exhibited the lowest thermal conductivity (0.79 Wm^−1^K^−1^ at 623 K), primarily due to increased phonon scattering caused by substitution-induced disorder. These results demonstrate the impact of CuFeSe_2−y_S_y_ solid solutions on thermoelectric performance by reducing thermal conductivity and enhancing electrical conductivity. Future work will focus on optimizing doping strategies to control the p–n transition and improve the power factor, ultimately enhancing ZT values.

## Data Availability

The original contributions presented in this study are included in the article; further inquiries can be directed to the corresponding author.
